# Effects of Dietary Lipid Levels on the Growth, Muscle Fatty Acid and Amino Acid Composition, Antioxidant Capacity, and Lipid Deposition in Mirror Carp (*Cyprinus carpio*)

**DOI:** 10.3390/ani14172583

**Published:** 2024-09-05

**Authors:** Xiaona Jiang, Zhenguo Song, Chitao Li, Xuesong Hu, Yanlong Ge, Lei Cheng, Xiaodan Shi, Zhiying Jia

**Affiliations:** 1 Heilongjiang River Fisheries Research Institute, Chinese Academy of Fishery Sciences, Harbin 150076, China; jiangxiaona@hrfri.ac.cn (X.J.); songzhenguo@hrfri.ac.cn (Z.S.); lichitao@hrfri.ac.cn (C.L.); huxuesong@hrfri.ac.cn (X.H.);; 2 Key Laboratory of Freshwater Aquatic Biotechnology and Breeding, Ministry of Agriculture and Rural Affairs, Harbin 150076, China

**Keywords:** mirror carp, dietary lipids, weight gain rate, serum lipid-related indices, DHA + EPA, antioxidant enzyme activity

## Abstract

**Simple Summary:**

Dietary lipids are the main source of energy for the development of fish larvae, and an adequate supply of essential fatty acids is essential for rapidly growing larvae. Mirror carp (*Cyprinus carpio)* has been cultivated widely because of its advantages of a fast growth rate, strong disease resistance, and high feed conversion rate. In this study, mirror carp larvae were fed iso-nitrogen (approximately 33% dietary protein) diets with different lipid levels (3%, 5%, 7%, 9%, 11%, and 13%), and the weight gain rate, serum lipid-related indices, muscle amino acid and fatty acid contents, liver lipid deposition, antioxidant enzyme activity, and expression of genes related to growth and fat metabolism were determined in different experimental groups. The optimal dietary lipid requirement of the mirror carp cultivar (6.86 ± 0.95 g) was 9%. This study provides data for improving the breeding of new common carp varieties with unsaturated fatty acids.

**Abstract:**

In fish, increasing the crude lipid level of feed can save protein and improve feed utilization. Mirror carp (*Cyprinus carpio*) is one of the most widely farmed fish species in the world. In this study, mirror carp larvae were fed isonitrogenous diets with different lipid levels (3%, 5%, 7%, 9%, 11%, and 13%). The rearing trial lasted for eight weeks. The results revealed that when the fat content was 9%, the AWGR, WGR, and FCR were highest, whereas FCR was lowest. The AWGR was correlated with the dietary lipid level, and the regression equation was y = −2.312x^2^ + 45.01x + 214.49. Compared with those in the control group, the T-CHO and TG contents were significantly greater in the 13% lipid content groups and significantly lower in the 9% lipid content groups (*p* < 0.05). In terms of muscle quality, the contents of MUFAs, PUFAs, and DHA + EPA were significantly greater than those in the other experimental groups (*p* < 0.05). Oil red O staining revealed a lipid content of 13% with severe fat deposition. In addition, the results of the analysis of antioxidant enzyme activity revealed that the activities of GSH, CAT and T-AOC were significantly greater at the 9% lipid content, and that the MDA content was significantly greater at the 13% lipid content (*p* < 0.05). Similarly, the mRNA levels of *GH*, *IGF-I*, *FAS*, and *LPL* were significantly highest at a lipid level of 9% (*p* < 0.05). The above results revealed that the optimal dietary lipid requirement for the fast growth of mirror carp (6.86 ± 0.95 g) was 9.74% on the basis of nonlinear regression analysis of the AWGR. The dietary lipid level (9%) improved the growth, stress resistance, and lipid utilization of mirror carp to a certain extent.

## 1. Introduction

With the rapid development of the aquaculture industry, problems such as protein source shortages and nutrient imbalances in diets have occurred. Many scholars have reported that fat can be used as a preferred energy source and that increasing the crude lipid level of feed can save protein and improve feed utilization [1,2,3]. Dietary lipids can provide cells with fatty acids, cholesterol, and phospholipids, which help cells maintain normal structure and function [4]. However, the need for and ability to use fat are limited by the physiological conditions of fish, and long-term consumption of high-fat feed causes fat metabolism disorders and a decrease in meat quality, which severely affects the health of fish [5,6]. As an important organ of lipid metabolism, the liver is very sensitive to dietary lipids. Previous studies have shown that a high-fat diet can lead to lipid accumulation and affect the metabolic function of the liver [7,8]. Previous studies have shown that appropriate lipid levels can promote growth and protein utilization, but excess lipids impair growth and lead to lipid accumulation, especially in the liver [9,10]. In addition, studies have shown that fish growth is related to the regulation of *growth hormone* (*GH*), *insulin-like growth factor I* (*IGF-I*) mRNA expression [11,12]. Fat deposition is also regulated by the expression of related genes, such as *lipoprotein lipase* (*LPL*) and *fatty acid synthase* (*FAS*) [13,14]. Thus, the lipid content of the feed must be set at a reasonable level to meet the needs of the fish. In addition, increasing lipid levels and appropriately reducing protein levels are effective ways to address the shortage of protein resources in the aquaculture industry.

Fish are good sources of highly unsaturated fatty acids (HUFAs) in the human diet. In fish, the lipids in the feed can both supply the energy of the fish and provide the essential fatty acids for the body. In general, the content of essential fatty acids in the muscle of freshwater fish is low, and it is more easily affected by changes in dietary lipid content [15]. In Nile tilapia (*Oreochromis niloticus*), muscles from which more fish oil was added to the experimental diet contained more DHA and EPA than did those from the control diet group [16]. Similar results were reported for Atlantic salmon (*Salmo salar*) [17]. These findings support the rationale of using sustainable raw materials rich in polyunsaturated fatty acids for feed production in fish.

Common carp are the third most widely farmed freshwater fish in the world and have extremely high economic value [18]. Mirror carp is an increasingly larger proportion of production due to its advantages of a fast growth rate, strong disease resistance, and high feed conversion rate [19,20]. To determine the optimal dietary lipid levels, this study investigated the effects of dietary lipid levels on the growth performance, unsaturated fatty acid content, liver lipid deposition, serum biochemical indices, antioxidant capacity, and expression of genes involved in lipid metabolism of the mirror carp. This study provides data for improving the breeding of new common carp varieties with unsaturated fatty acids.

## 2. Materials and Methods

### 2.1. Experimental Diets

The feed formula used in this study was designed according to the feed formula of common carp (GB/T 36782-2018, 2019) [21]. In the diet, fish meal was the main protein source, and fish oil and soybean oil were the main fat sources. Six different diets with 3%, 5%, 7%, 9%, 11%, 13%, and 15% lipid content with approximately 33% dietary protein were fed to the mirror carp. The lipid gradient was regulated with microcrystalline cellulose (Table 1). The solid material was ground into powder and mixed, and fish oil, soybean oil, and water were added. Particles with a diameter of 2 mm were produced by a laboratory pellet machine. After the particles were dried at 60 °C for 5 h, all the feed was stored in plastic-lined bags at −20 °C.

### 2.2. Experimental Design and Feeding Management

All animal procedures in this study were conducted according to the guidelines for the care and use of laboratory animals of the Heilongjiang River Fisheries Research Institute of the Chinese Academy of Fishery Sciences (CAFS). The studies involving animals were reviewed and approved by the Committee for the Welfare and Ethics of Laboratory Animals of the Heilongjiang River Fisheries Research Institute, CAFS (approval code: 2019-03-15). The experimental fish used in this study were one-year-old mirror carp, which were obtained from the Kuandian Fisheries Experiment Station of Heilongjiang River Fisheries Research Institute of the Chinese Academy of Fishery Sciences. Before the experiment, the mirror carp were temporarily raised for two weeks and fed commercial feed at the appropriate feed level so that they could fully adapt to the feeding environment. Healthy mirror carp (*n* = 216, 6.86 ± 0.95 g) were randomly placed into 18 aquariums (50 cm × 50 cm × 20 cm) and 6 experimental groups, with three replicates in each group. The rearing trial lasted for eight weeks. The feeding amount was 3% of the body weight (3 times a day). The water was changed three times a day (1/3 of the aquarium volume), and the temperature difference during the water change was controlled within 2 °C.

### 2.3. Sample Collection

After the trial and fasting for 24 h, fish anesthetic (MS-222, 100 mg/L; Beijing Green Hengxing Biological Technology Co., Beijing, China) was used to anaesthetize the experimental fish. The body weights of 12 common carp from each experimental group were measured, 9 of which were selected for tissue collection. Blood was collected from the tail vein, placed in a centrifuge tube, kept at 4 °C for 1–2 h, and centrifuged at 3500 r/min for 10 min. The upper serum was drawn, dispensed into centrifuge tubes, and placed at −20 °C for use in the determination of serum biochemical indicators. The liver and intestine were collected from the fish, mixed with samples, and placed in a −80 °C freezer for the determination of corresponding indicators.

### 2.4. Fish Performance

In this study, the relative weight gain rate (WGR), absolute weight gain rate (AWGR), protein efficiency ratio (PER), and feed conversion ratio (FCR) were used to evaluate the growth performance of each experimental group. The WGR rates and AGRw rates were calculated according to the following formulas:

WGR (g/g) = (Wx + 1 − Wx)/Wx;(1)

AWGR (g/d) = (Wx + 1 − Wx)/(tx + 1 − tx);(2)

PER (%) = 100 × (Wx + 1 − Wx)/P(3)

FCR (%) = F/(Wx + 1 − Wx)(4)

In the above formula, Wx represents the initial body weight (g), Wx + 1 represents the final body weight (g), t represents the interval (d) between the two sampling periods, P represents the intake of feed crude protein content (g), and F represents the dry weight of feed intake (g).

### 2.5. Indicator Determination

The approximate composition of the experimental fish pellet diet and muscle were assessed according to the standard procedure of the AOAC [22]. A vacuum freeze dryer (FD-1A-50, Yuming, Beijing, China) was used to determine the muscle moisture content of the experimental fish. The crude protein content in the muscle of the experimental fish was determined via the Kjeldahl method (GB 5009.5) [23]. The Soxhlet extraction method (GB5009.6) [24] was used to determine the crude lipid content of the muscle in the experimental fish. Chromatography (1260 and 7890 A, Agilent, Santa Clara, CA, USA) was used to determine the amino acid composition in the muscle, with tryptophan determined by alkaline hydrolysis (laboratory method) and other amino acids determined by acid hydrolysis (GB5009.124) [25]. The fatty acid contents were determined via gas chromatography–mass spectrometry (7890B-5977A, Agilent, Santa Clara, CA, USA). After the intestinal and liver tissues were statically ground and mixed with normal saline (1:9) in a low-temperature environment, the supernatants were assayed for intestinal digestive enzyme activities and liver antioxidant indicators. The crude ash content was determined by burning the sample to a constant weight at 550 °C. Liver antioxidant indices, including total protein (TP, A045-2, Coomassie brilliant blue method), lysodeikticus (CAT, A007-1-1, ammonium molybdate method), total antioxidant capacity (T-AOC, A015-2-1, ABTS method) activity glutathione (GSH, A006-2-1, microplate method), and malondialdehyde (MDA, A003-1, thiobarbituric acid method) content, were determined via enzyme activity detection kits (Jiancheng, Nanjiang, China). Serum biochemical indicators, including TP (105-000451-00), total cholesterol (T-CHO) (105-000448-00), triglyceride (TG, 105-000449-00), high-density lipoprotein (HDL, 105-000463-00), low-density lipoprotein (LDL, 105-000464-00), and glucose (GLU, 105-000949-00), which were purchased from Mindray (Shenzhen, China) were determined via immunoturbidimetry, and all indicators were measured via a biochemical analyzer (BS350E, Mindray, Shenzhen, China). There were 9 fish in each experimental group in this study.

### 2.6. Oil Red O Stain

The liver tissues of each group of experimental fish were fixed with 4% paraformaldehyde, dehydrated with sucrose, and frozen slices (longitudinal cutting) were prepared via a frozen slicing mechanism (Cryostar Nx50, Thermo Fisher Scientific, Waltham, MA, USA). Afterward, the samples were allowed to dry at room temperature for approximately 15 min. The sections were stained with saturated oil red O solution for 8–10 min (in the dark, covered). The mixture was differentiated with two cylinders of 60% isopropyl alcohol for approximately 10 s and then gently soaked in distilled water. The slices were removed, incubated for 3 s, dipped in hematoxylin for 3–5 min, and soaked in 3 tanks of pure water for 5 s, 10 s, and 30 s, respectively. The differentiation solution was differentiated for 2–8 s, distilled water was added for 10 s in each tank, and blue solution was added for 1 s in each tank. The slices were gently immersed in the tap water of the two tanks for 5 s and 10 s, and the staining effect was examined via microscopy (BX53, Thermo Fisher Scientific, Waltham, MA, USA).

### 2.7. RNA Isolation and Real-Time Polymerase Chain Reaction (RT–PCR)

RNA was extracted from the liver tissue of all the fish. Total RNA was extracted from common carp tissues via the RNeasy Mini Kit (Qiagen, Hilden, Germany) according to the manufacturer’s instructions. The integrity and quality of the RNA were analyzed via 1.5% agarose gel electrophoresis. The purity of the RNA was determined via UV spectrophotometry. The OD260:280 ratio for all the RNA samples was between 1.8 and 2.0. cDNA was synthesized from 1 µg of total RNA via the PrimeScript™ RT Reagent Kit with the gDNA Eraser (TaKaRa, Beijing, China) according to the manufacturer’s instructions. Specific primers (Table 2) were obtained via Primer Premier 5.0. RT–qPCR was performed according to the TB Green™ Premix Ex Taq™ II (TaKaRa, Beijing China) instructions via an ABI7500 system (Life Technologies, Carlsbad, CA, USA). The primer specificity was confirmed by dissociation curve analysis. *Beta-actin* (*β-actin*) was used as an internal reference gene. *β-actin* has been reported to be the most suitable reference gene in mirror carp [26,27], and its expression remained highly stable across the samples. Double-distilled water was used instead of the template as the negative control. The relative gene expression levels of *GH*, *IGF-I*, *FAS*, and *LPL* were determined via the 2 ^(−ΔΔCT)^ method [28]. The primers used in this study are shown in Supplementary Table S1. At least three replicates per experimental group were used.

### 2.8. Data Analysis

The data are presented as the means ± SDs of at least three replicates. The differences in the trial parameters among the fish fed different test diets were analyzed via one-way analysis of variance (ANOVA). Multiple comparisons utilizing Duncan’s test were performed on the variables if significant differences were detected. Analysis of the experimental data was performed via IBM SPSS software (version 22.0, IBM Corp., Armonk, New York, NY, USA), and *p* < 0.05 was considered statistically significant. Moreover, the relationships between the growth performance AWGR and dietary lipid levels (3%, 5%, 7%, 9%, 11%, and 13%) was examined via nonlinear regression. All of the data were checked for a normal distribution by a one-sample Kolmogorov–Smirnov test, and homogeneity of variances was assessed via Levene’s test. All the experiments were performed at least three times.

## 3. Results

### 3.1. Growth Performance

**Figure 1:**
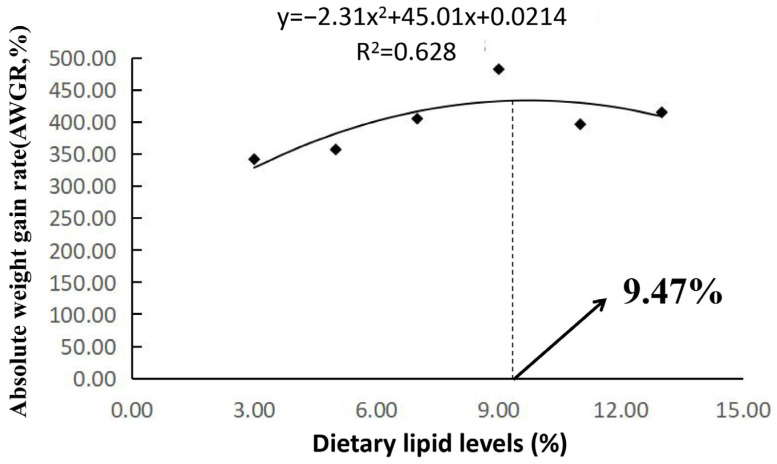
Relationships between AWGR (y) and dietary lipid level (x) for mirror carp larvae (*Cyprinus carpio*) fed the test diets for 8 weeks. The regression equation was y = −2.312x^2^ + 45.01x + 214.49 (R^2^ = 0.63, *p* < 0.05).

The growth performance of the mirror carp fed different lipid concentrations was determined, and the results are shown in Table 2. The WGR and AWGR were the highest when the lipid content was 9%, and they were lower when the lipid content was 3% or 5%. The AWGR rate of the dietary lipid content at 9% was significantly greater than that of the other five experimental groups, and the WGR was significantly greater than that at 3% and 5% (*p* < 0.05). Furthermore, the WGR and AWGR in the 5% and 3% groups were significantly lower than those in the other experimental groups (*p* < 0.05). When the lipid content was 9%, the PER was significantly greater, and the FCR was significantly lower (*p* < 0.05). The survival rate of all the experimental groups was 100%. The AWGR was correlated with the dietary lipid level, and the regression equation was y = −2.312x^2^ + 45.01x + 214.49 (R^2^ = 0.63, *p* < 0.05; Figure 1).

### 3.2. Serum Biochemical Indices

The serum biochemical indices of the different experimental groups were analyzed (Table 3). The T-CHO contents of the 13% and 11% experimental groups were significantly greater than those of the other four experimental groups, and the TG content of the 13% experimental group was significantly greater than those of the 3%, 5%, 7%, and 9% groups (*p* < 0.05). With increasing dietary lipid content, the HDL content first increased and then decreased, and its content in the 9% experimental group was significantly greater than that in the other experimental groups, whereas that in the 11% and 13% experimental groups was significantly greater than that in the 3%, 5%, and 7% groups (*p* < 0.05).

### 3.3. Basic Nutrients

The essential nutrients, amino acid components, and fatty acid contents of the muscle in the different experimental groups were determined (Table 4, Table 5 and Table 6). The results revealed that the crude fat content of the muscle in the 9% lipid group was significantly greater than that in the other groups (*p* < 0.05) (Table 4). The muscle crude fat content decreased significantly as the dietary lipid level increased from 9% to 13% (*p* < 0.05). The contents of EAA in the muscle of the 7% and 9% lipid content groups were significantly greater than those in the 5% and 11% lipid content groups (*p* < 0.05) (Table 5). The FAA content at the 11% lipid content was significantly lower than that in the other experimental groups (*p* < 0.05). In addition, the highest lipid content of ∑EAA/∑TAA was 9%. The contents of TFA, SFA, MUFA, PUFA, and DHA + EPA in the muscle of the 9% lipid content group were significantly greater than those in the other lipid groups (*p* < 0.05) (Table 6).

### 3.4. Oil Red O Staining

**Figure 2:**
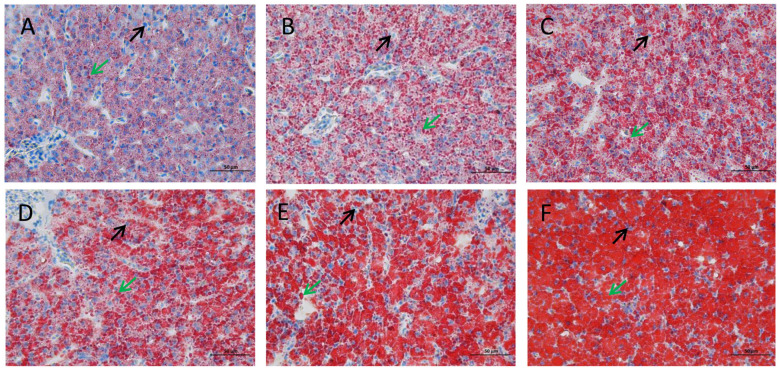
Liver samples of different lipid levels in the diet were stained with Oil Red O (400×). (**A**) 3%; (**B**) 5%; (**C**) 7%; (**D**) 9%; (**E**) 11%; (**F**) 13%. The small blue spots are nuclei (black arrows); the red nearly spherical spots are lipid droplets (green arrows).

The liver tissues of the experimental groups with different lipid levels were stained with Oil Red O, which stained the lipids and nuclei red and blue, respectively (Figure 2). The nuclei of the six lipid-level groups were partially pressed to the side of the cell and were partially located in the center of the cell. Compared with those with 3% lipid content, the size and number of the lipid droplets in the experimental groups with 5%, 7%, 9%, 11%. and 13% lipid contents were greater. In addition, the 13% lipid group had the largest red areas with severe fat deposition, and the 3% lipid group had the smallest red areas.

### 3.5. Antioxidant Oxidase Content

**Figure 3:**
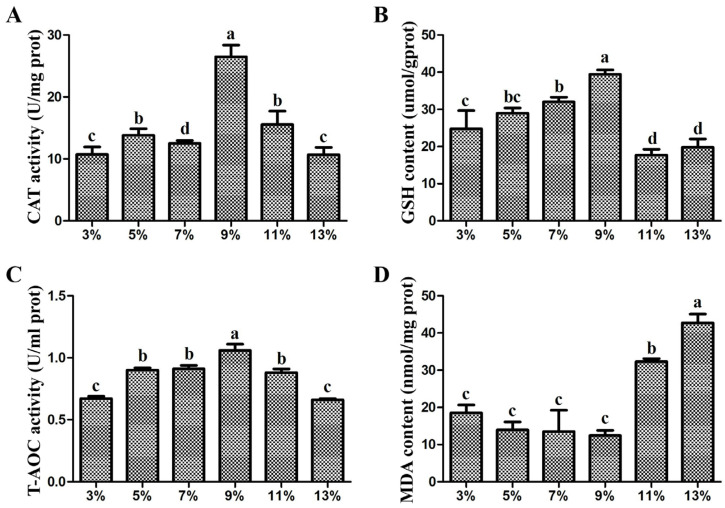
Antioxidant capacity of different lipid levels in the diet. Antioxidant enzymes, including (**A**) CAT (U/mg prot), (**B**) GSH (umol/g prot), (**C**) T-AOC (U/ml), and (**D**) MDA (nmol/mg prot), were assayed in the liver. The same row with different letters indicates significant differences between groups according to one-way ANOVA (*p* < 0.05).

The indices of the antioxidant enzymes in the liver tissues of the different lipid levels in the experimental groups were analyzed (Figure 3). With increasing lipid levels, the activities of GSH, CAT, and T-AOC first increased and then decreased, while the MDA content showed the opposite trend. The activities of GSH, CAT, and T-AOC in the 9% lipid content group were significantly greater than those in the other groups, while the content of MDA was significantly lower (*p* < 0.05).

### 3.6. Expression of Genes Related to Growth and Lipid Synthesis

**Figure 4:**
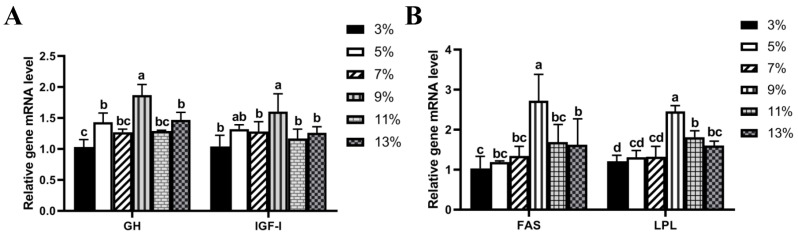
Effect of dietary lipid levels on *GH*, *IGF-I,* and *FAS* and *LPL* mRNA expression in liver tissue from *Cyprinus carpio*. The relative expression of was evaluated via the relative fold value compared with that of the 3% lipid content. (**A**) *GH* and *IGF-I*; (**B**) *FAS* and *LPL*. Lowercase letters indicate significant effects on the relative gene expression of growth-related genes (*p* < 0.05).

The relative expression levels of *GH*, *IGF-I*, *FAS,* and *LPL* in the liver tissue were determined at different lipid levels in liver tissue (Figure 4). The mRNA expression levels of *GH* and *IGF-I* first increased and then decreased with increasing dietary lipid levels. When the lipid content was 9%, the *GH* gene expression level was significantly greater, and the relative expression level of *IGF-I* was significantly greater than that in the 3%, 7%, 11%, and 13% groups (*p* < 0.05). The results revealed that the mRNA expression of *FAS* and *LPL* also tended to first increase and then decreased with increasing dietary lipid content. The relative expression levels of *FAS* and *LPL* were significantly greater when the lipid content was 9% (*p* < 0.05).

## 4. Discussion

Previous studies have shown that appropriate lipid levels can promote growth and protein utilization, but excess lipids can impair growth and lead to lipid accumulation in fish [5,9]. This study revealed that the WGR and AWGR were the highest when the lipid content was 9%, and they were significantly greater than those when the lipid content was 3% or 5% (*p* < 0.05). Furthermore, when the lipid content was 9%, the PER was significantly greater, and the FCR was significantly lower (*p* < 0.05). The results indicated that the AWGR and WGR were the highest when the dietary lipid content was 9%, and either too low or too high of a lipid content affected the growth rate of the mirror carp, which is consistent with the results for common carp [29], grass carp (*Ctenopharyngodon idella*) [10], and kelp grouper (*Epinephelus bruneus*) [6]. The optimum dietary lipid content of mirror carp was 9.74%, according to the regression analysis of the AWGR. The analysis of serum biochemical indices can provide valuable information about the physiology and health of fish [30]. Lipids are a class of organic molecules, including triglycerides and sterols, that are crucial in energy storage and cell signaling [31]. TG and T-CHO are important components of blood lipids and are synthesized mainly in the liver. When blood lipid levels in the body are too high, there may be a variety of conditions, such as hypertriglyceridemia [32]. T-CHO is a sterol that is a precursor to steroid hormones and bile acids [33]. The results of this study revealed that the serum TG and T-CHO contents significantly increased with increasing dietary lipid content (13%), indicating that the consumption of high-lipid diets can increase the serum lipid content, which may induce a variety of diseases. These results were similar to those reported for Siberian sturgeon (*Acipenser baerii*) [34] and hybrid sturgeon (*Acipenser baerii* × *Acipenser gueldenstaedtii*) [35]. HDL is a key anti-atherosclerosis lipoprotein that transfers cholesterol in the blood from peripheral cells to the liver and has been linked to a reduced risk of coronary heart disease and lipid accumulation [36,37]. When the lipid content was 9%, the HDL content was significantly greater than that of the other experimental groups (*p* < 0.05), indicating that the risk of mirror carp disease could be reduced when the dietary lipid content was 9%, and the risk of disease increased when the dietary lipid content was too high. In addition, this study revealed that when the dietary lipid content was not less than 9%, the HDL content was high, indicating that a high-lipid diet increased the level of HDL and accelerated the transport of cholesterol to the liver. This result is similar to those of previous studies [38,39,40].

Muscle nutrient composition is an important factor affecting the nutritional value of fish [41]. The results revealed that the crude lipid content in the muscle of the 9% lipid content group was significantly greater than that in the other experimental groups, indicating that the dietary lipid content could affect the muscle lipid content of the mirror carp. At present, the protein and amino acid contents in muscle affect the nutritional value, flavor and function of fish, and amino acids in fish play a key role in maintaining human health [42,43]. The contents of EAA, FAA, and ∑EAA/∑TAA in the muscle of the 9% lipid content group were greater than those in the other groups, indicating that the dietary lipid content can affect the nutritional value of muscle, and that the muscle of the 9% lipid content group had greater nutritional value than the other lipid level groups. Fish meat is the main source of unsaturated fatty acids for humans. The fatty acid composition and lipid content in muscle are important parameters for evaluating the nutritional quality and flavor of fish [44]. PUFAs are essential for maintaining cell membrane structure and function [45], whereas EPA and DHA in PUFAs have antioxidant and antiaging effects, which can prevent the occurrence of cardiovascular diseases and other diseases in mammals and promote brain development [46,47,48,49]. In this study, the contents of PUFA and total DHA + EPA in the muscle of the 9% lipid content group were significantly greater than those in the other groups, but the SFA content was also significantly greater than that in the other groups (*p* < 0.05), which might be related to the high crude lipid content.

Liver tissue is an important metabolic tissue [50]. Feeding fish an excess amount of lipids can lead to lipid deposition in liver tissue [9]. With increasing lipid content, the lipid deposition in the liver was obvious, especially in the 13% experimental group, indicating that feeding a high-lipid diet leads to lipid deposition in the liver of the mirror carp. However, the activities of GSH, CAT, and T-AOC are usually used to evaluate the effects of dietary lipids on the antioxidant capacity of tissues and participation in the process of removing reactive oxygen species, while the MDA content is positively correlated with ROS accumulation in vivo [51,52,53,54]. From the perspective of antioxidants, with increasing lipid content, the activities of GSH, CAT, and T-AOC first tended to increase and then tended to decrease, and the highest and lowest activities were observed at the 9% lipid content and 13% lipid content, respectively, whereas the trend in the MDA content was the opposite. These findings suggest that the dietary lipid content can significantly affect the antioxidant capacity of the liver, and that the antioxidant capacity was optimal when the lipid content was 9%, whereas the antioxidant capacity was low at the 13% lipid content, which might also be the reason for the low weight gain rate of the high-lipid content groups of the mirror carp.

Muscle is the main edible part for the consumer, and its growth is regulated by a variety of genes. In fish, *IGF* and insulin-like growth factor-binding proteins subsequently act on *IGFR* on the cell surface, promoting cell proliferation and growth [11,12]. Previous studies have found that liver tissue detection of *IGF-I* and *GH* gene expression levels is reliable and can reflect the growth status of fish [55,56,57]. The relative expression levels of *GH* and *IGF-I* first increased and then decreased with increasing dietary lipid levels. When the lipid content was 9%, the mRNA expression of *GH* and *IGF-I* was the highest, which was similar to the result for the weight gain rate, indicating that the growth-related genes in muscle could be used as indicators to evaluate the growth of the mirror carp. Furthermore, *LPL* and *FAS* promote adipogenesis, which can lead to fat accumulation, whereas the overfeeding of lipids can alter the developmental programming of pathways involved in lipid metabolism in liver tissue [13,14,58]. Our results revealed that the relative expression levels of *FAS* and *LPL* temporarily increased with increasing dietary lipid content, which suggested that differences in dietary lipid content significantly affected fat accumulation in the livers of common carp. Furthermore, our results revealed that the relative expression of *FAS* and *LPL* subsequently decreased with increasing dietary lipid content (lipid contents greater than 9%), which confirmed that a high-fat diet inhibited the secretion of FAS and LPL in mirror carp. It was speculated that the lipid levels in diets could change lipid utilization during the ontogenesis of mirror carp. This result has also been reported in Nile tilapia (*Oreochromis niloticus*) [59] and zebrafish (*Danio rerio*) [58], as well as in dark barbel catfish (*Pelteobargrus vachelli*) [60], but the specific inhibition mechanism still needs to be further determined.

Thus, the present study revealed that dietary lipid levels had significant effects on the growth, muscle quality, serum biochemical indices, liver lipid deposition, and antioxidant enzyme activity of the mirror carp. The optimal dietary lipid requirement of 6.86 ± 0.95 g was 9%. This study provides a research basis for the breeding of new varieties of high-quality carp and provides solutions for the shortage of protein sources and the imbalance of feed nutrition.

## 5. Conclusions

In conclusion, the optimal dietary lipid requirement for the fast growth of mirror carp (6.86 ± 0.95 g) should be 9.74% on the basis of nonlinear regression analysis of the AWGR. The dietary lipid level (9%) improved the growth, stress resistance, and lipid utilization of mirror carp to a certain extent.

## Figures and Tables

**Table 1:** Composition of the experimental diet (air-dry matter, %).

Ingredient	Lipid Content					
	3%	5%	7%	9%	11%	13%
Rapeseed meal ^1^	11.20	11.20	11.20	11.20	11.20	11.20
Soybean meal ^1^	37.00	37.00	37.00	37.00	37.00	37.00
Fish meal ^1^	15.00	15.00	15.00	15.00	15.00	15.00
Soybean oil	0.55	1.57	2.60	3.61	4.64	5.65
Fish oil	0.55	1.58	2.59	3.62	4.63	5.66
Cornstarch	7.00	7.00	7.00	7.00	7.00	7.00
Wheat middling ^1^	15.00	15.00	15.00	15.00	15.00	15.00
Dicalcium phosphate	1.50	1.50	1.50	1.50	1.50	1.50
Microcrystalline cellulose	10.30	8.25	6.21	4.17	2.13	0.09
Vitamin premix ^2^	0.50	0.50	0.50	0.50	0.50	0.50
Trace mineral premix ^2^	0.50	0.50	0.50	0.50	0.50	0.50
Choline chloride	0.30	0.30	0.30	0.30	0.30	0.30
Methionine	0.30	0.30	0.30	0.30	0.30	0.30
Threonine	0.30	0.30	0.30	0.30	0.30	0.30
Total	100.00	100.00	100.00	100.00	100.00	100.00
Proximate composition						
Crude protein	32.76	32.87	33.11	33.09	33.12	33.24
Crude lipid	3.07	4.93	7.09	9.05	10.91	13.10
Total Phosphorus	1.26	1.25	1.23	1.28	1.26	1.24
Lysine	1.97	1.94	1.98	1.95	1.93	1.97

Note: ^1^ Proximate composition: rapeseed meal (crude protein, 38%; crude lipid, 3.8%); soybean meal (crude protein, 46%;crude lipid, 1.5%), fish meal (crude protein, 65%; crude lipid, 4.85%); wheat middling (crude protein, 13%; crude lipid, 1.2%). ^2^ Proximate composition: vitamin premix (VA, 8000IU; VB1, 15 mg; VB2, 30 mg; VB6, 10 mg; VB12, 1 mg; VC, 100 mg; VD3, 3000IU; VE, 100 mg; VK3, 5 mg); trace mineral premix (nicotinamide, 175 mg; d-biotin, 2 mg; inositol, 800 mg; folic acid, 6 mg; pantothenic acid, 50 mg; Cu, 3 mg; Fe, 30 mg; Mn, 13 mg; I, 0.8 mg; Zn, 65 mg).

**Table 2:** Effects of dietary lipid levels on the growth performance of *Cyprinus carpio*.

	Lipid Content					
	3%	5%	7%	9%	11%	13%
Initial body weight (g)	7.09 ± 0.19	7.17 ± 0.39	6.89 ± 0.08	6.52 ± 0.40	6.95 ± 0.30	6.77 ± 0.26
Final body weight (g)	31.33 ± 4.14 ^a^	32.67 ± 0.21 ^b^	34.81 ± 0.22 ^ab^	37.74 ± 3.07 ^ab^	34.50 ± 2.85 ^ab^	34.82 ± 3.03 ^ab^
Absolute weight gain rate (AWGR, %)	341.89 ± 24.32 ^c^	357.01 ± 25.52 ^c^	405.06 ± 8.84 ^b^	482.15 ± 83.72 ^a^	396.36 ± 36.93 ^b^	414.94 ± 54.17 ^b^
Relative weight gain rate (WGR, g/d)	0.47 ± 0.01 ^b^	0.46 ± 0.01 ^b^	0.50 ± 0.01 ^ab^	0.56 ± 0.06 ^a^	0.49 ± 0.05 ^ab^	0.50 ± 0.06 ^ab^
Protein efficiency ratio (PER,%)	1.51 ± 0.00 ^c^	1.49 ± 0.10 ^c^	1.60 ± 0.02 ^c^	2.18 ± 0.06 ^a^	1.92 ± 0.20 ^b^	1.67 ± 0.03 ^c^
Feed conversion ratio (FCR,%)	2.87 ± 0.1 ^a^	2.56 ± 0.19 ^b^	2.12 ± 0.03 ^c^	1.26 ± 0.03 ^e^	1.65 ± 0.17 ^d^	1.61 ± 0.03 ^d^
Survival rate/%	100	100	100	100	100	100

Note: Data are presented as the mean ± SDs (*n* = 3). The same row with different letters indicates significant differences between groups on the basis of one-way ANOVA (*p* < 0.05).

**Table 3:** Effects of dietary lipid levels on the serum biochemical parameters of *Cyprinus carpio* (mmol/L).

	Lipid Content					
	3%	5%	7%	9%	11%	13%
Total cholesterol (T-CHO)	3.64 ± 0.36 ^b^	3.38 ± 0.23 ^b^	3.50 ± 0.39 ^b^	3.45 ± 0.91 ^b^	4.07 ± 0.21 ^a^	4.32 ± 0.55 ^a^
Triglyceride (TG)	0.76 ± 0.17 ^b^	0.65 ± 0.12 ^b^	0.65 ± 0.10 ^b^	0.74 ± 0.15 ^b^	0.93 ± 0.12 ^ab^	1.29 ± 0.57 ^a^
High-density lipoprotein (HDL-C)	1.76 ± 0.26 ^c^	1.85 ± 0.36 ^c^	1.98 ± 0.07 ^c^	2.43 ± 0.32 ^a^	2.14 ± 0.29 ^b^	2.15 ± 0.29 ^b^
Low-density lipoprotein (LDL-C)	0.96 ± 0.15	0.9 ± 0.13	0.87 ± 0.10	0.92 ± 0.20	0.91 ± 0.11	0.95 ± 0.12
Glucose (GLU)	3.99 ± 0.58	4.94 ± 0.18	4.05 ± 0.33	4.25 ± 0.81	4.89 ± 0.65	4.09 ± 0.54

Note: Data are presented as the mean ± SD (*n* = 9). The same row with different letters indicates significant differences between groups on basis of one-way ANOVA (*p* < 0.05).

**Table 4:** Effect of dietary lipid levels on the muscular proximate chemical composition of *Cyprinus carpio* (wet weight, g/100 g).

Lipid Content	Moisture	Crude Ash	Crude Fat	Crude Protein
3%	77.07 ± 0.85 ^a^	0.82 ± 0.04 ^b^	2.97 ± 0.21 ^e^	18.13 ± 0.71
5%	75.23 ± 0.49 ^b^	0.83 ± 0.04 ^b^	3.53 ± 0.15 ^d^	19.40 ± 0.78
7%	74.60 ± 0.26 ^b^	1.07 ± 0.06 ^a^	4.60 ± 0.40 ^b^	18.67 ± 0.32
9%	72.57 ± 0.49 ^c^	1.07 ± 0.06 ^a^	6.20 ± 0.20 ^a^	19.20 ± 0.72
11%	74.33 ± 0.96 ^b^	1.03 ± 0.06 ^a^	4.57 ± 0.12 ^b^	19.03 ± 0.90
13%	74.30 ± 0.80 ^b^	1.03 ± 0.06 ^a^	4.03 ± 0.15 ^c^	19.27 ± 0.71

Note: Data are presented as the mean ± SD of three replicates (*n* = 9). The same row with different letters indicates significant differences between groups on basis of one-way ANOVA (*p* < 0.05).

**Table 5:** Effect of dietary lipid levels on the amino acid content of *Cyprinus carpio* (g/100 g, dry weight).

	Lipid Content					
	3%	5%	7%	9%	11%	13%
Aspargine (Asp) *	1.513 ± 0.032 ^a^	1.543 ± 0.061 ^a^	1.587 ± 0.097 ^a^	1.530 ± 0.087 ^a^	1.290 ± 0.069 ^b^	1.470 ± 0.096 ^a^
Throsine (Thr) ^#^	0.650 ± 0.035 ^ab^	0.610 ± 0.030 ^b^	0.710 ± 0.050 ^a^	0.683 ± 0.042 ^ab^	0.480 ± 0.036 ^c^	0.627 ± 0.075 ^ab^
Serine (Ser) ^●^	0.533 ± 0.035 ^ab^	0.573 ± 0.035 ^a^	0.593 ± 0.040 ^a^	0.557 ± 0.047ab	0.497 ± 0.031 ^b^	0.563 ± 0.021 ^ab^
Glutamine (Glu) *	2.047 ± 0.095 ^a^	1.983 ± 0.112 ^a^	2.180 ± 0.161 ^a^	2.137 ± 0.153 ^a^	1.610 ± 0.122 ^b^	1.973 ± 0.156 ^a^
Glycine (Gly) *	0.640 ± 0.010 ^ab^	0.643 ± 0.042 ^ab^	0.710 ± 0.078 ^a^	0.720 ± 0.046 ^a^	0.577 ± 0.021 ^b^	0.653 ± 0.032 ^ab^
Alanine (Ala) *	0.900 ± 0.010 ^a^	0.910 ± 0.044 ^a^	0.940 ± 0.066 ^a^	0.923 ± 0.0513 ^a^	0.813 ± 0.040 ^b^	0.903 ± 0.040 ^a^
Cystine (Cys) ^●^	0.112 ± 0.050	0.089 ± 0.018	0.107 ± 0.006	0.117 ± 0.023	0.084 ± 0.023	0.105 ± 0.030
Valine (Val) ^#^	0.640 ± 0.199 ^ab^	0.410 ± 0.036 ^cd^	0.707 ± 0.083 ^ab^	0.757 ± 0.021 ^a^	0.310 ± 0.020 ^d^	0.517 ± 0.150 ^bc^
Methionine (Met) ^#^	0.377 ± 0.021 ^a^	0.380 ± 0.010 ^a^	0.387 ± 0.025 ^a^	0.357 ± 0.038 ^ab^	0.317 ± 0.045 ^b^	0.383 ± 0.032 ^a^
Isoleucine (Ile) ^#^	0.567 ± 0.171 ^ab^	0.360 ± 0.200 ^cd^	0.617 ± 0.086 ^ab^	0.677 ± 0.021 ^a^	0.2533 ± 0.0252 ^d^	0.440 ± 0.140 ^bc^
Leucine (Leu) ^#^	1.183 ± 0.062 ^a^	1.123 ± 0.058 ^a^	1.260 ± 0.092 ^a^	1.2367 ± 0.064 ^a^	0.930 ± 0.061 ^b^	1.133 ± 0.107 ^a^
Tyrosine (Tyr) ^●^	0.457 ± 0.025	0.483 ± 0.031	0.463 ± 0.038	0.447 ± 0.046	0.433 ± 0.040	0.457 ± 0.021
Phenylalanine(Phe) ^#^	0.597 ± 0.040 ^a^	0.563 ± 0.015 ^a^	0.627 ± 0.038 ^a^	0.617 ± 0.038 ^a^	0.490 ± 0.027 ^b^	0.560 ± 0.056 ^a^
Lysine (Lys) ^#^	1.397 ± 0.145 ^ab^	1.250 ± 0.063 ^b^	1.493 ± 0.117 ^a^	1.490 ± 0.078 ^a^	1.023 ± 0.067 ^c^	1.310 ± 0.181 ^ab^
Histidine (His) ^※^	0.550 ± 0.020 ^bc^	0.610 ± 0.020 ^a^	0.627 ± 0.038 ^a^	0.587 ± 0.015 ^ab^	0.507 ± 0.012 ^c^	0.537 ± 0.038 ^c^
Arginine (Arg) ^※^	0.853 ± 0.055 ^ab^	0.790 ± 0.061 ^b^	0.927 ± 0.076 ^a^	0.923 ± 0.055 ^a^	0.657 ± 0.047 ^c^	0.827 ± 0.090 ^ab^
Proline (Pro) ^●^	0.513 ± 0.021 ^ab^	0.533 ± 0.035 ^a^	0.533 ± 0.038 ^a^	0.483 ± 0.021 ^ab^	0.410 ± 0.036 ^c^	0.467 ± 0.040 ^bc^
Tryptophan (Trp) ^#^	13.529 ± 0.836 ^a^	12.856 ± 0.616 ^a^	14.467 ± 1.036 ^a^	14.241 ± 0.802a	10.680 ± 0.653 ^b^	12.925 ± 1.271 ^a^
Total amino acids (TAA)	5.100 ± 0.130 ^a^	5.080 ± 0.254 ^a^	5.417 ± 0.385 ^a^	5.310 ± 0.311 ^a^	4.290 ± 0.251 ^b^	5.000 ± 0.320 ^a^
Flavour amino acid (FAA)	5.410 ± 0.661 ^ab^	4.697 ± 0.218 ^b^	5.800 ± 0.448 ^a^	5.817 ± 0.298 ^a^	3.803 ± 0.266 ^c^	4.970 ± 0.737 ^ab^
Essential amino acid (EAA)	1.403 ± 0.074 ^ab^	1.400 ± 0.078 ^ab^	1.553 ± 0.117 ^a^	1.510 ± 0.070 ^ab^	1.163 ± 0.040 ^c^	1.363 ± 0.123 ^b^
Half essential amino acids (HEAA)	6.076 ± 0.151 ^a^	6.116 ± 0.291 ^a^	6.403 ± 0.430 ^a^	6.194 ± 0.423 ^a^	5.137 ± 0.328 ^b^	5.938 ± 0.385 ^a^
Nonessential amino acids (NEAA)	37.757	39.513	37.444	37.285	40.172	38.774
FAA/TAA (F/T, %)	39.887	36.536	40.086	40.855	35.599	38.328
EAA/TAA (E/T, %)	10.377	10.887	10.736	10.6079	10.904	10.554
HEAA/TAA%	88.981	76.807	90.582	93.983	74.021	83.398

Note: * represents flavor amino acids, # represents essential amino acid, ※ represents half essential amino acids, and ● is nonessential amino acid (*n* = 9). The same row with different letters indicates significant differences between groups on basis of one-way of ANOVA (*p* < 0.05).

**Table 6:** Effect of the dietary lipid level on the fatty acid content of *Cyprinus carpio* (g/100 g, dry weight).

	Lipid Content					
	3%	5%	7%	9%	11%	13%
C14:0	0.038 ± 0.003 ^c^	0.050 ± 0.006 ^c^	0.104 ± 0.012 ^b^	0.144 ± 0.012 ^a^	0.108 ± 0.015 ^b^	0.117 ± 0.009 ^b^
C15:0	0.006 ± 0.001 ^c^	0.008 ± 0.001 ^c^	0.016 ± 0.002 ^b^	0.022 ± 0.002 ^a^	0.016 ± 0.002 ^b^	0.017 ± 0.009 ^b^
C16:0	0.590 ± 0.060 ^e^	0.695 ± 0.035 ^de^	0.997 ± 0.118 ^b^	1.247 ± 0.048 ^a^	0.849 ± 0.075 ^c^	0.804 ± 0.047 ^cd^
C17:0	0.009 ± 0.001 ^c^	0.011 ± 0.001 ^c^	0.018 ± 0.002 ^b^	0.024 ± 0.002 ^a^	0.016 ± 0.002 ^c^	0.016 ± 0.001 ^c^
C18:0	0.189 ± 0.016 ^c^	0.204 ± 0.006 ^c^	0.264 ± 0.018 ^b^	0.337 ± 0.017 ^a^	0.206 ± 0.010 ^c^	0.194 ± 0.014 ^c^
C20:0	0 ^b^	0 ^b^	0.008 ± 0.001 ^a^	0.012 ± 0.002 ^a^	0.012 ± 0.008 ^a^	0.008 ± 0.001 ^a^
Saturated fatty acid (SFA)	0.833 ± 0.082 ^c^	0.968 ± 0.048 ^c^	1.408 ± 0.145 ^b^	1.787 ± 0.076 ^a^	1.207 ± 0.099 ^b^	1.155 ± 0.071 ^b^
C16:1	0.106 ± 0.009 ^c^	0.123 ± 0.008 ^c^	0.198 ± 0.028 ^b^	0.261 ± 0.012 ^a^	0.180 ± 0.025 ^b^	0.168 ± 0.011 ^b^
C18:1n9c	0.970 ± 0.093 ^cd^	1.135 ± 0.036 ^c^	1.457 ± 0.136 ^b^	1.758 ± 0.135 ^a^	1.060 ± 0.114 ^cd^	0.912 ± 0.079 ^d^
C20:1	0.060 ± 0.007 ^c^	0.0812 ± 0.003 ^c^	0.156 ± 0.022 ^b^	0.222 ± 0.024 ^a^	0.147 ± 0.017 ^b^	0.161 ± 0.008 ^b^
C22:1n9	0.007 ± 0.001 ^d^	0.001 ± 0.0012 ^cd^	0.015 ± 0.003 ^b^	0.018 ± 0.002 ^a^	0.012 ± 0.001 ^bc^	0.015 ± 0.002 ^b^
C24:1	0.007 ± 0.001 ^c^	0.007 ± 0.001 ^c^	0.012 ± 0.0014 ^b^	0.017 ± 0.002 ^a^	0.012 ± 0.003 ^b^	0.013 ± 0.001 ^b^
Monounsaturated fatty acid (MUFA)	1.152 ± 0.112 ^c^	1.356 ± 0.043 ^c^	1.837 ± 0.186 ^b^	2.276 ± 0.171 ^a^	1.411 ± 0.155 ^c^	1.269 ± 0.099 ^c^
C18:2n6c *	0.441 ± 0.042 ^c^	0.689 ± 0.079 ^c^	0.689 ± 0.0584 ^b^	0.728 ± 0.094 ^a^	0.411 ± 0.041 ^c^	0.374 ± 0.030 ^c^
C18:3n3 *	0.043 ± 0.002 ^c^	0.071 ± 0.009 ^a^	0.063 ± 0.006 ^b^	0.084 ± 0.008 ^a^	0.049 ± 0.005c	0.048 ± 0.004 ^c^
C20:2	0.014 ± 0.002 ^c^	0.184 ± 0.001 ^b^	0.018 ± 0.003 ^b^	0.024 ± 0.002 ^a^	0.014 ± 0.001 ^c^	0.014 ± 0.001 ^c^
C20:3n3	0.022 ± 0.004 ^ab^	0.025 ± 0.001 ^a^	0.019 ± 0.0007 ^b^	0.025 ± 0.003 ^a^	0.014 ± 0.001 ^c^	0.012 ± 0.000 ^c^
C20:3n6	0 ^d^	0.004 ± 0.000 ^c^	0.005 ± 0.001 ^b^	0.007 ± 0.000 ^a^	0.005 ± 0.001 ^b^	0.007 ± 0.000 ^a^
C20:4n6	0.035 ± 0.005 ^e^	0.062 ± 0.004 ^d^	0.125 ± 0.020 ^c^	0.203 ± 0.022 ^a^	0.143 ± 0.012 ^bc^	0.164 ± 0.011 ^b^
C20:5n3 Eicosapentaenoic acid (EPA)	0.063 ± 0.007 ^d^	0.069 ± 0.005 ^d^	0.127 ± 0.005 ^c^	0.180 ± 0.016 ^a^	0.130 ± 0.009 ^bc^	0.147 ± 0.012 ^b^
C22:6n3 Docosahexaenoic acid (DHA)	0.164 ± 0.0198 ^c^	0.171 ± 0.012 ^c^	0.307 ± 0.011 ^b^	0.452 ± 0.042 ^a^	0.314 ± 0.011 ^b^	0.345 ± 0.012 ^b^
Polyunsaturated fatty acid (PUFA)	0.781 ± 0.080 ^c^	1.100 ± 0.106 ^b^	1.241 ± 0.101 ^b^	1.702 ± 0.176 ^a^	1.080 ± 0.077 ^b^	1.110 ± 0.067 ^b^
DHA + EPA	0.226 ± 0.027 ^d^	0.231 ± 0.016 ^d^	0.435 ± 0.015 ^c^	0.632 ± 0.057 ^a^	0.444 ± 0.019 ^bc^	0.491 ± 0.024 ^b^
Essential fatty acid (EFA)	0.484 ± 0.042 ^c^	0.760 ± 0.088 ^a^	0.641 ± 0.065 ^b^	0.812 ± 0.101 ^a^	0.459 ± 0.045 ^c^	0.422 ± 0.033 ^c^
Total fatty acid (TFA)	2.767 ± 0.264 ^d^	3.423 ± 0.179 ^c^	4.487 ± 0.385 ^b^	5.763 ± 0.422 ^a^	3.697 ± 0.326 ^c^	3.533 ± 0.231 ^c^

Note: * represents the essential fatty acids. The same row with different letters indicates significant differences between groups on basis of one-way ANOVA (*p* < 0.05) (*n* = 9).

## Data Availability

Data will be made available upon request.

## Supplementary Materials

The following supporting information can be downloaded at: https://www.mdpi.com/article/10.3390/ani14172583/s1, Table S1: All primers used in this experiment.
