# Supplementary material for: Effects of Dietary Lipid Levels on the Growth, Muscle Fatty Acid and Amino Acid Composition, Antioxidant Capacity, and Lipid Deposition in Mirror Carp (Cyprinus carpio)
Source: Animals (Basel). 2024 Sep 5;14(17):2583. doi: 10.3390/ani14172583 (PMC11394664; doi:10.3390/ani14172583)
Supplement: Supplementary file 1 [file animals-14-02583-s001.zip › animals-3153431-supplementary.pdf]

Supplementary Table S1. All primers used in this experiment.

Gene	Primers	Sequence 5'-3'
Gh	F	TCAAGGGATGTCTCGATGGT
	R	CTACAGGGTGCAGTTGGAAT
Igf-1	F	GGGCCTAGTTCAAGACGG
	R	AGTGGCTTTGTCCAGGTAA
Fas	F	GACAGGCCGCTATTGCTATT
	R	TGCCGTAAGCTGAGGAAATC
Lpl	F	CGCTCCATTCACCTGTTCAT
	R	GCTGAGACACATGCCCTTATT
β-actin	F	GGCAGGTCATCACCATCGG
	R	TTGGCATACAGGTCTTTACGG
